# Bioremediation of *n*-alkanes, polycyclic aromatic hydrocarbons, and heavy metals from wastewater using seaweeds

**DOI:** 10.1007/s11356-023-29549-8

**Published:** 2023-09-15

**Authors:** Faiza M. A. Akl, Suzan I. Ahmed, Mostafa M. El-Sheekh, Mofida E. M. Makhlof

**Affiliations:** 1 Department of Biological and Geological Sciences, Faculty of Education, Alexandria University, Alexandria, Egypt; 2 Botany Department, Faculty of Science, Tanta University, Tanta, 31527 Egypt; 3 Botany and Microbiology Department, Faculty of Science, Damanhour University, Damanhour, Egypt

**Keywords:** *n*-Alkanes, Polycyclic romatic hydrocarbons, Heavy metals, Marine macroalgae, Adsorption

## Abstract

The removal of *n*-alkanes, polycyclic aromatic hydrocarbons, and heavy metals from wastewater using three dried seaweeds *Ulva intestinalis* Linnaeus (green alga), *Sargassum latifolium* (Turner) C.Agardh (brown alga), and *Corallina officinalis* Kützing (red alga) has been shown to evaluate their potential usage as inexpensive adsorbents. Under natural environmental conditions, numerous analytical methods, including zeta potential, energy dispersive X-ray spectroscopy (EDX), SEM, and FT-IR, are used in this study. The results showed that *n*-alkanes and polycyclic aromatic hydrocarbons adsorption increased with increasing contact time for all three selected algae, with a large removal observed after 15 days, while the optimal contact time for heavy metal removal was 3 h. *S. latifolium* dry biomass had more potential as bioadsorbent, followed by *C. officinalis* and then *U. intestinalis*. *S. latifolium* attained removal percentages of 65.14%, 72.50%, and 78.92% for light *n*-alkanes, heavy *n*-alkanes, and polycyclic aromatic hydrocarbons (PAHs), respectively, after 15 days. Furthermore, it achieved removal percentages of 94.14, 92.62, 89.54, 87.54, 82.76, 80.95, 77.78, 73.02, and 71.62% for Mg, Zn, Cu, Fe, Cr, Pb, Cd, Mn, and Ni, respectively, after 3 h. Carboxyl and hydroxyl from FTIR analysis took part in wastewater treatment. The zeta potentials revealed that algal cells have a negatively charged surface, and the cell surface of *S. latifolium* has a more negative surface charge than *U. intestinalis* and C. *officinalis*. Our study suggests that seaweeds could play an important role in wastewater treatment and thus help as an economical, effective, and ecofriendly bioremediation system for ecological health and life protection.

## Introduction

Water is extremely important; thus, there is a constant desire for its quality to be improved and preserved. With the increase of the industrial world and the global population, environmental issues have also gotten worse. Water pollution is a typical form of pollution that the globe is now dealing with. Overexploitation and deterioration of natural water supplies are the results of the rise in global population over the past century. (UNESCO 2021; Blanco-Vieites et al. 2022). Industrial activities are responsible for 23% of total water consumption (Blanco-Vieites et al. 2022). Our water resources’ quality is getting worse every day as a result of the persistent introduction of harmful substances into them. (Huang et al. 2021). Industrialization, civilization, agriculture, and other environmental and global changes are the primary causes of water contamination. (Haiba 2019; Ahmad et al. 2021). Even still, 80% of the wastewater that has not been cleaned is discharged into freshwater bodies used for domestic reasons. This gives rise to global water stress because of the increasing scarcity of freshwater resources. According to a study, by the year 2025, around 60% of the world’s population will experience water stress. (Khalid et al. 2018). Heavy metals, polycyclic aromatic hydrocarbons (PAHs), and a wide variety of *n*-alkanes can be found in wastewater particularly that is discharged by different types of industries (Haiba 2019; Redha 2020).

Water resources have been discovered to be contaminated by a few hundred organic contaminants such as alkanes and polycyclic aromatic hydrocarbons. Due to their numerous negative impacts and cancer-causing nature, these contaminants are extremely harmful. (Xia et al. 2022). Due to their noxiousness and abundance in the environment, the Environmental Protection Agency (EPA) has named 16 unsubstituted PAHs as the most aggressive pollutants (Qari and Hassan 2017). Urban areas are filled with PAHs, which are widely dispersed throughout the environment, either spontaneously (as components of crude oil) or due to man activity (like petroleum industries). Due to their cytotoxic characteristics, PAHs are particularly concerning (mutagenesis and carcinogenesis) (Haiba et al. 2019; Tomar and Jajoo 2021) and they can easily enter the food chain; this, in turn, negatively affects human health (Kottuparambil and Agusti 2020; Premnath et al. 2021).

Heavy metals are a type of water contaminants that pose a great threat to the ecological system (Redha 2020; Tang et al. 2023). Basically, the danger of heavy metals to all human life aspects lies in their non-degradable property and a very long biological half-life; thus, their bioaccumulation throughout the food chain causes significant consequences on higher trophic levels. (Zhang et al. 2019; Nathana et al. 2022). In addition, the toxicity of heavy metals also occurs in case of relatively low concentrations (Rehman et al. 2018). It has been found that exposure to toxic quantities of these substances significantly contributes to the deterioration of defense mechanisms and raises the chance of developing cancer (Yuan et al. 2019). Additionally, heavy metals make organic pollutants less biodegradable, prolonging their half-life in the environment and intensifying other toxic wastes’ impacts (Briffa et al. 2020).

Given the seriousness of these pollutants (*n*-alkanes, PAHs, and heavy metals), an immediate environmental issue that needs to be resolved is the efficient removal of them from wastewater. Traditional methods for removing these pollutants are mainly divided into chemical and physical methods. Despite the high cost, these techniques offer their benefits, requiring high amounts of energy, and can easily produce secondary pollution problems due to the formation of toxic by-products. Moreover, these techniques can be ineffectual when applied to low quantities of heavy metals because their effectiveness relies on concentration. (Shrestha et al. 2021; Zeng et al. 2022). Alternative methods to traditional ones for removing heavy metals from wastewater include biosorption. (Blanco-Vieites et al. 2022). Biosorption technology uses biological materials as adsorbents to clear the water bodies from a wide range of contaminants like aliphatic and aromatic hydrocarbons, pesticides, dyes, and heavy metal contaminates (Esfandiar et al. 2021; Dubey et al. 2023). Biosorption technology is simple, effective, more sustainable, ecofriendly, and low-cost. It has a promising future for development and is consistent with the countries’ present environmental protection philosophy. It is also a popular subject in recent studies (Cheng et al. 2019; Kumar et al. 2019). Bacteria, fungi, yeast, algae, and other microorganisms are the main types of materials that can be employed as biosorbents for bioremediation and the accumulation of organic pollutants and heavy metals (Chen et al. 2020). Because of its porous structure, substantial surface area, widespread dispersion, and other valuable aspects, algae have a promising removal action on various pollutants in water. Algae can be used as a straightforward and inexpensive sorbent for various contaminants from contaminated water (Rocha et al. 2020; Banerjee 2021; Chu et al. 2022). The comparative cost analysis showed that algae-based treatment is 10 times less expensive than traditional methods of treating industrial wastewater containing heavy metals. In addition, valuable metal ions like gold and silver can be recovered using algae (Jaafari and Yaghmaeian 2019).

Egypt’s industrial and population growth is swift and turbulent (Maghraby and Hassan 2021). The wastewater from Amia drain at the El-Tabia region in Alexandria, Egypt, consists of irrigation drainage and wastewater from the different industries, where this region hosts over 25 industrial activities. The present study explored the performance of *Ulva intestinalis* L*.* (green alga), *Sargassum latifolium* (Turner) C.Agardh (brown alga), and *Corallina officinalis* Kützing (red alga), in the removal of *n*-akanes, polycyclic aromatic hydrocarbons, and heavy metals from Amia drain water under natural climatic conditions. The work intends to create a naturally occurring, economically advantageous, and technically workable method for cleansing contaminated water resources. And it is worth saying that for the first time, the utilization of dried seaweeds as bioadsorbents for *n*-alkanes, polycyclic aromatic hydrocarbons, and heavy metal removal from wastewater under natural environmental conditions is done. Furthermore, this is the first work to study the removal of *n*-alkanes as individual hydrocarbons using dry seaweeds and study this large number of PAHs, in addition to the use of FTIR, SEM, EDX, and Zeta potential analyses to explain the ability of seaweeds to remove *n*-alkanes and PAHs.

## Materials and methods

### Algal species and sampling

*Ulva intestinalis* Linnaeus (green alga) and *Corallina officinalis* Kützing (red alga) were collected from Abu Qir in Alexandria, while *Sargassum latifolium* (Turner) C.Agardh (brown alga) was collected from the Gulf of Suez, Egypt coast; all samples were identified according to the methods of Aleem (1978), Aleem (1991), and Lipkin and Silva (2002) and be sure using the Algae Base website (Guiry 2020). Following collection, whole algae were thoroughly rinsed with seawater multiple times to remove any sand that may have become attached. Rhizoidal sections were also removed to prevent microbial infection. Algal materials were brought to the lab in plastic bags that contained saltwater. They are open-air dried for 24 h and oven dried for 3 h at 60 °C. Following fine grinding of the dried samples, they were stored in plastic bags at room temperature.

### Wastewater sampling

**Fig. 1:**
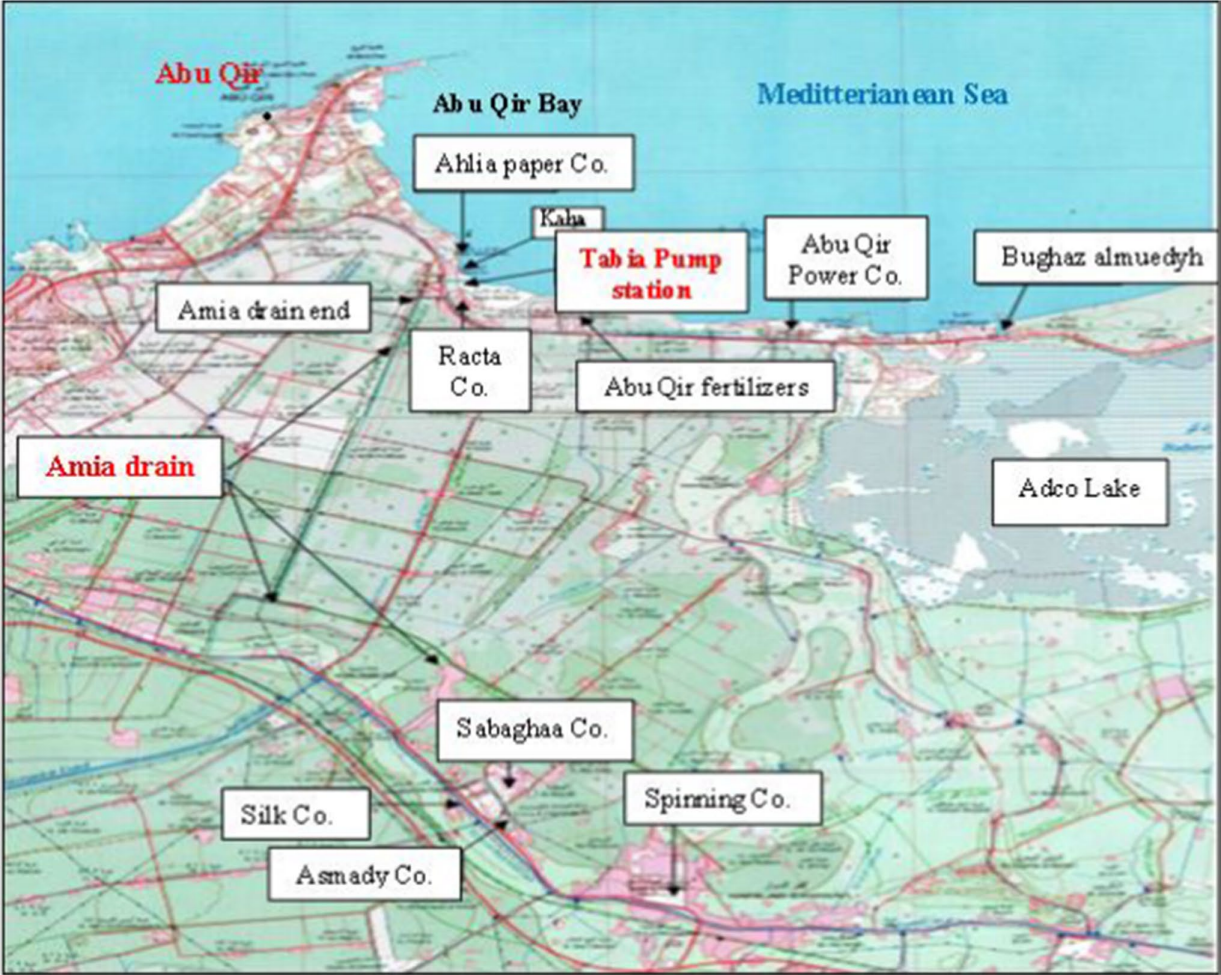
El Amia drain at El-Tabia Region in Alexandria

Wastewater samples for culturing were collected from El Amia drain at El-Tabia pumping stations (Fig. 1) and discharged into Abu Qir Bay in Alexandria. Water samples were collected in empty n-hexane or dichloromethane glass bottles (not previously used for other purposes), and their composition is shown in Tables 1, 2, 3, and 4. These samples were taken to the laboratory without any filtering and kept in a cool, dark environment to preserve their properties.

**Table 1:** Wastewater heavy metal composition

Heavy metals	Cd	Cr	Cu	Fe	Mg	Mn	Ni	Pb	Zn
Initial conc. mg/l	0.027	0.029	1.329	0.618	11.081	0.063	0.074	0.042	5.662

**Table 2:** Concentration of light *n*-alkanes (µg/l) remained in wastewater at different time intervals using *Ulva intestinales* L*.* dry biomass

Hydrocarbon conc. (µg/l)				
Hydrocarbon	Time (days)			
	Initial conc	5th day	10th day	15th day
C_13_	6.92 ± 0.49	6.32 ± 0.42	5.11 ± 0.31	3.03 ± 0.23
C_14_	6.45 ± 0.46	6.02 ± 0.38	4.34 ± 0.22	3.04 ± 0.22
C_15_	5.35 ± 0.36	5.15 ± 0.33	4.11 ± 0.20	2.62 ± 0.19
C_16_	4.96 ± 0.29	3.88 ± 0.21	3.46 ± 0.19	2.37 ± 0.15
C_17_	4.88 ± 0.25	4.46 ± 0.23	3.42 ± 0.18	2.12 ± 0.12
Pristane	3.92 ± 0.22	3.04 ± 0.17	2.44 ± 0.14	2.20 ± 0.13
C_18_	5.82 ± 0.37	4.64 ± 0.22	4.07 ± 0.21	3.63 ± 0.19
Phytane	6.55 ± 0.39	5.82 ± 0.34	4.23 ± 0.22	3.11 ± 0.19
C_19_	3.43 ± 0.21	3.33 ± 0.18	2.94 ± 0.14	1.38 ± 0.17
C_20_	2.84 ± 0.18	2.65 ± 0.15	1.89 ± 0.12	1.53 ± 0.12
∑Light *n*-alkanes	51.12 ± 4.25	45.31 ± 3.54	36.01 ± 3.24	25.03 ± 2.13

**Table 3:** Concentration of heavy *n*-alkanes (µg/l) remained in wastewater at different time intervals using *Ulva intestinalis* L. dry biomass

Hydrocarbon conc. (µg/l)				
Hydrocarbon	Time (days)			
	Initial conc. conc	5th day	10th day	15th day
C_21_	2.77 ± 0.19	2.52 ± 0.15	2.34 ± 0.18	2.03 ± 0.14
C_22_	2.71 ± 0.19	2.62 ± 0.16	2.42 ± 0.16	2.11 ± 0.18
C_23_	3.38 ± 0.27	2.85 ± 0.17	2.39 ± 0.18	2.15 ± 0.20
C_24_	4.86 ± 0.22	3.26 ± 0.19	3.17 ± 0.19	2.34 ± 0.22
C_25_	5.67 ± 0.33	4.43 ± 0.24	3.38 ± 0.20	2.62 ± 0.29
C_26_	5.45 ± 0.31	3.67 ± 0.19	3.25 ± 0.18	2.23 ± 0.25
C_27_	4.71 ± 0.23	4.04 ± 0.20	3.96 ± 0.19	3.18 ± 0.23
C_28_	5.61 ± 0.33	5.13 ± 0.27	4.02 ± 0.22	3.21 ± 0.21
C_29_	5.21 ± 0.26	5.14 ± 0.22	3.84 ± 0.20	2.24 ± 0.17
C_30_	5.15 ± 0.26	4.93 ± 0.20	4.82 ± 0.25	3.58 ± 0.21
C_31_	6.89 ± 0.35	6.38 ± 0.31	4. 97 ± 0.23	2.81 ± 0.19
C_32_	5.67 ± 0.32	3.69 ± 0.19	2.23 ± 0.18	1.64 ± 0.16
C_33_	4.94 ± 0.25	4.63 ± 0.21	2.94 ± 0.20	1.74 ± 0.15
C_34_	4.92 ± 0.22	3.22 ± 0.18	2.69 ± 0.19	1.57 ± 0.16
C_35_	3.90 ± 0.19	2.72 ± 0.19	2.59 ± 0.16	1.50 ± 0.14
C_36_	2.86 ± 0.17	1.97 ± 0.17	1.58 ± 0.14	1.12 ± 0.12
∑Heavy *n*-alkanes	74.7 ± 5.12	61.2 ± 4.98	45.62 ± 3.72	36.07 ± 3.22

**Table 4:** Concentration of polycyclic aromatic hydrocarbons (µg/l) remained in wastewater at different time intervals using *Ulva intestinales* L*.* dry biomass

Polycyclic aromatic hydrocarbons conc. (µg/l)				
PAHs	Time (days)			
	Initial conc	5th day	10th day	15th day
Naphthalene	4.28 ± 0.29	3.92 ± 0.28	2.38 ± 0.19	1.73 ± 0.18
Flurene	2.47 ± 0.26	2.37 ± 0.22	1.89 ± 0.16	1.25 ± 0.12
Phenanthrene	5.82 ± 0.31	4.71 ± 0.28	2.54 ± 0.20	1.86 ± 0.19
Benzo (a) anthracene	2.52 ± 0.19	2.34 ± 0.19	2.04 ± 0.19	1.12 ± 0.11
Anthracene	2.29 ± 0.17	2.27 ± 0.20	1.82 ± 0.16	1.21 ± 0.14
Pyrene ∑ PAHs	3.87 ± 0.27	2.42 ± 0.22	2.01 ± 0.19	1.36 ± 0.19
∑PAHs	21.16 ± 2.75	18.04 ± 2.58	12.68 ± 2.48	8.23 ± 2.42

### Adsorption experiments

Experiments were conducted in 1-l beakers by mixing 3 g of dry algal mass with 1 l of wastewater. Beakers had earlier been rinsed with HNO_3_. The mixtures were periodically stirred for the necessary amount of time with aeration. Ten ml water were removed from the beakers after 1, 2, 3, 4, and 5 h then filtered by filter paper, and the residues (algal materials) are returned to the beakers to complete the experiment, while the filtrate metal content was determined using atomic absorption spectrophotometer measurements. Sampling was done after 5, 10, and 15 days to determine the ultimate concentration of hydrocarbons in filtrates (Wrabel and Peckol 2000; Akl and Ahmed 2022). The temperature was 25 °C and all studies were conducted in a natural climate.

Heavy metal and hydrocarbon removal rate (Re%) were determined as follows based on the beginning and end concentrations of each compound in the effluent (Tsekova et al. 2010):

$$\mathrm{Re}\%=({C}_{0}-{C}_{x})/{C}_{0}\times 100$$

where *C*_0_ and *C*_*x*_ are the concentrations of initial and final given pollutant in wastewater (mg/l^−1^), respectively. All trials were done in triplicates with the mean and standard error calculation.

### Analytical procedures for wastewater

Analysis of the tested *n*-alkanes and polycyclic aromatic hydrocarbons (PAHs) in wastewater was performed according to Alkio et al. (2005). The extraction in a Teflon stopcock-supplied separating funnel was done for a duplicate measured volume of wastewater samples (1 l each) by 5 min shaking with 30 ml dichloromethane. Extraction was repeated three times. After the complete separation between the aqueous and organic phases, over the collection flask, the organic phase was passed through granular anhydrous sodium sulfate placed in filter paper. The organic extracts were combined in a 100-ml Erlenmeyer flask. Using a rotary evaporator, the extract was concentrated to a volume of around 5 ml, where the water bath temperature was maintained at around 40 °C, then to the final evaporation (for drying) by using a gentle stream of clean and dry nitrogen.

The extracted residue was re-solved in n-hexane in a GC vial fitted with a screw cap lined with Teflon; the final volume of extract was adjusted at 0.5 ml to inject GC–MS spectrometer (Thermo Scientific ISQ 2009) and helium at 1 ml/min as the carrier gas. The use was made of the TG-1MS column, composed of 100% dimethyl polysiloxane, fused silica, 30 m, 0.32-mm item diameter (i.d.), and 0.25-m film thickness. After 3 min of splitless injection mode, the injection port temperature reached 250 °C. Next, split mode with a split ratio of 1:100 was used. The temperature of the column was programmed to rise from 80 to 240 °F at a rate of 7 °C/min, then to 300 °F at a rate of 3 °C/min, and to maintain that temperature for 5 min (Alkio et al. 2005).

### FTIR

FTIR was carried out through KBr pellets as the test substance at 4 cm^*−*1^ resolution and 400–4000 cm^*−*1^ range to demonstrate all potential functional groups on the algal biomass surface. The wave number (cm^*−*1^) on the Y axis and transmittance (%) on the X axis were used to plot the generated peaks.

### SEM and EDX

A scanning electron microscope was applied to inspect the surface of the algal biomass both before and after the adsorption process (JSM 6490 LV, JEOL, Ltd., Tokyo, Japan). After samples had dried naturally on a double-sided carbon tape, they were photographed using various magnifications. Magnifications ranging from 15,000 to 35,000 nm and scanning voltages of 20 to 30 kV were used to measure the sizes. Using the same device, an energy dispersive X-ray (EDX) analysis of the component content and elemental distribution in algal biomasses between 0 and 12 keV was performed (Rajkumar et al. 2020).

### Zeta potential (ζ)

The algal biomass effective surface charges at various variables were evaluated using a zeta analyzer (ZetaPlus, Brookhaven Instruments, Holtsville, NY, USA) at a range from − 200 to + 200 mV. A total of 25 g of algal biomass sample water dilution was done ten times. The sample was then run at 20 Hz for 15 min in a sonicator. Algal biomass was diluted to stop it from aggregating. (Basant et al. 2021).

## Results and discussion

### Assessment of the efficiencies of *Ulva intestinalis *L. dry biomass for the removal of light n-alkanes, heavy *n*-alkanes, and polycyclic aromatic hydrocarbons (PAHs)

**Fig. 2:**
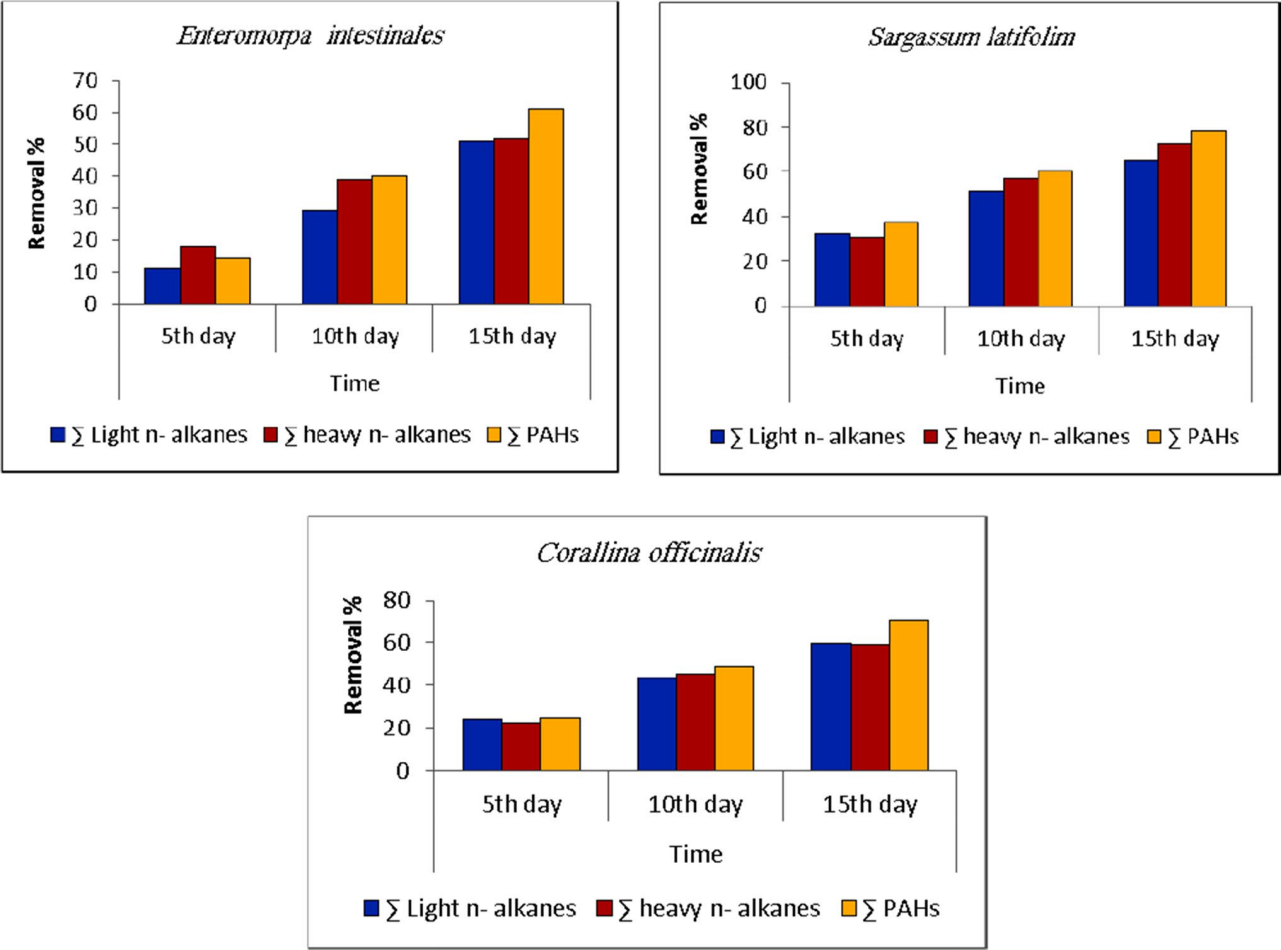
Removal percentage of light *n*-alkanes, heavy *n*-alkanes, and polycyclic aromatic hydrocarbons (PAHs) from wastewater at different time intervals using *Ulva intestinalis*, *Sargassum latifolium*, and *Corallina officinalis* dry biomass

Adsorption of light *n*-alkanes, heavy *n*-alkanes, and PAHs by *U. intestinalis* dry biomass at different time intervals was shown in Tables 2, 3, and 4. Hydrocarbon adsorption by this alga increased with the increase of contact time since a large decrease was observed after 15 days, where the concentration of light *n*-alkanes, heavy *n*-alkanes, and PAHs decreased from 51.12, 74.7, and 21.16 µg/l in initial concentration samples to 25.03, 36.07, and 8.23 µg/l, respectively, which represent half their values compared to the initial concentration. The alga achieved removal percentages 51.03%, 51.71%, and 61.10% for light *n*-alkanes, heavy *n*-alkanes, and PAHs, respectively, after 15 days (Fig. 2). This agrees with El-Shoubaky and Mohammad (2016) who reported that gasoline bioaccumulation differed significantly using the two algal species *Ulva lactuca* and *Enteromorpha clathrate*.

The supply and quality of water are thought to be seriously impacted by the rise in global pollution, industrialization, and rapid economic growth. Industrial, agricultural, and municipal wastewater are the three main sources of this waste, and they all contain pollutants such as xenobiotics, microplastics, regular alkanes, polycyclic aromatic hydrocarbons, and heavy metals (Ahmad et al. 2021; Bhatt et al. 2022; Ofrydopoulou et al. 2022).

The alternative biological agent abundantly found in nature algae is highlighted in this study as a potential sink for removing such hazardous compounds from water (Agüera et al. 2020; Ahmed et al. 2022; Sánchez et al. 2022; Satya et al. 2023). In order to clean and cleanse water, less dangerous chemicals can be used if algae are used. Algal biomass has also removed heavy metals and hydrocarbons in wastewater treatment facilities. The algae either store, absorb, or metabolize these harmful substances into significant levels (Ahmad et al. 2022; Alazaiza et al. 2022; Ghodrati et al. 2022).

### Assessment of the efficiencies of *Sargassum latifolium* (Turner) C.Agardh dry biomass for removal of light *n*-alkanes, heavy n-alkanes, and polycyclic aromatic hydrocarbons (PAHs)

In the same trend, *S. latifolium* dry biomass showed a sharp decline in hydrocarbons concentration until the 15 days, where light *n*-alkanes decreased from 51.12 to 17.82 µg/l. Heavy *n*-alkanes decreased from 74.7 to 20.54 µg/l, and PAHs decreased from 21.16 to 4.46 µg/l (Tables 5, 6, and 7). The alga achieved removal percentages of 65.14%, 72.50%, and 78.92% for light *n*-alkanes, heavy *n*-alkanes, and PAHs, respectively, after 15 days (Fig. 2). Wrabel and Peckol (2000) reported that the analysis of treatment samples of oil spills showed a complete loss of *n*-alkanes after 18 days focusing the brown alga *Fucus vesiclosus*.

**Table 5:** Concentration of light *n*-alkanes (µg/l) remained in wastewater at different time intervals using *Sargassum latifolium* dry biomass

Hydrocarbon conc. (µg/l)				
Hydrocarbon	Time (days)			
	Initial conc. conc	5th day	10th day	15th day
C_13_	6.92 ± 0.49	4.22 ± 0.27	3.09 ± 0.18	2.04 ± 0.16
C_14_	6.45 ± 0.46	4.02 ± 0.21	3.12 ± 0.21	2.02 ± 0.18
C_15_	5.35 ± 0.36	4.11 ± 0.23	2.02 ± 0.15	1.96 ± 0.15
C_16_	4.96 ± 0.29	3.62 ± 0.19	3.11 ± 0.19	2.11 ± 0.21
C_17_	4.88 ± 0.25	3.13 ± 0.19	2.21 ± 0.16	1.89 ± 0.17
Pristane	3.92 ± 0.22	2.84 ± 0.18	2.23 ± 0.17	1.78 ± 0.17
C_18_	5.82 ± 0.37	4.24 ± 0.25	2.94 ± 0.20	2.11 ± 0.20
Phytane	6.55 ± 0.39	4.73 ± 0.29	3.02 ± 0.22	1.52 ± 0.29
C_19_	3.43 ± 0.21	2.26 ± 0.17	2.13 ± 0.16	1.16 ± 0.19
C_20_	2.84 ± 0.18	1.25 ± 0.18	1.20 ± 0.15	1.09 ± 0.13
∑Light *n*-alkanes	51.12 ± 4.25	34.42 ± 3.14	24.99 ± 2.98	17.82 ± 2.51

**Table 6:** Concentration of heavy *n*-alkanes (µg/l) remained in wastewater at different time intervals using *Sargassum latifolium* dry biomass

Hydrocarbon conc. (µg/l)				
Hydrocarbon	Time (days)			
	Initial conc. conc	5th day	10th day	15th day
C_21_	2.77 ± 0.19	2.11 ± 0.15	1.86 ± 0.18	1.32 ± 0.19
C_22_	2.71 ± 0.19	2.13 ± 0.18	1.79 ± 0.20	1.14 ± 0.17
C_23_	3.38 ± 0.27	2.24 ± 0.19	1.63 ± 0.19	1.32 ± 0.19
C_24_	4.86 ± 0.22	3.27 ± 0.24	2.15 ± 0.19	1.21 ± 0.18
C_25_	5.67 ± 0.33	3.86 ± 0.28	2.23 ± 0.19	1.27 ± 0.20
C_26_	5.45 ± 0.31	3.92 ± 0.26	2.31 ± 0.22	2.09 ± 0.16
C_27_	4.71 ± 0.23	3.54 ± 0.22	2.81 ± 0.21	2.15 ± 0.21
C_28_	5.61 ± 0.33	4.03 ± 0.29	3.23 ± 0.22	1.01 ± 0.15
C_29_	5.21 ± 0.26	3.69 ± 0.24	2.45 ± 0.18	1.04 ± 0.15
C_30_	5.15 ± 0.26	3.46 ± 0.23	2.36 ± 0.15	1.22 ± 0.20
C_31_	6.89 ± 0.35	4.92 ± 0.31	3. 04 ± 0.20	1.24 ± 0.22
C_32_	5.67 ± 0.32	3.11 ± 0.26	2.64 ± 0.19	1.20 ± 0.20
C_33_	4.94 ± 0.25	4.35 ± 0.30	2.12 ± 0.16	1.11 ± 0.19
C_34_	4.92 ± 0.22	3.45 ± 0.29	2.02 ± 0.18	1.12 ± 0.15
C_35_	3.90 ± 0.19	2.68 ± 0.21	1.22 ± 0.14	1.06 ± 0.13
C_36_	2.86 ± 0.17	2.04 ± 0.16	1.13 ± 0.12	1.04 ± 0.13
∑Heavy *n*-alkanes	74.7 ± 5.12	52.8 ± 4.36	31.95 ± 3.55	20.54 ± 2.44

**Table 7:** Concentration of polycyclic aromatic hydrocarbons (µg/l) remained in wastewater at different time intervals using *Sargassum latifolium* dry biomass

Polycyclic aromatic hydrocarbons conc. (µg/l)				
PAHs	Time (days)			
	Initial conc	5th day	10th day	15th day
Naphthalene	4.28 ± 0.29	3.06 ± 0.26	1.38 ± 0.19	0.93 ± 0.16
Flurene	2.47 ± 0.26	1.98 ± 0.25	1.32 ± 0.16	0.62 ± 0.12
Phenanthrene	5.82 ± 0.31	2.68 ± 0.22	2.02 ± 0.19	1.04 ± 0.19
Benzo (a) anthracene	2.52 ± 0.19	2.03 ± 0.23	1.23 ± 0.22	0.48 ± 0.14
Anthracene	2.29 ± 0.17	1.73 ± 0.21	1.22 ± 0.20	0.54 ± 0.15
Pyrene ∑ PAHs	3.87 ± 0.27	1.84 ± 0.22	1.21 ± 0.19	0.85 ± 0.19
∑PAHs	21.16 ± 2.75	13.32 ± 2.48	8.38 ± 1.12	4.46 ± 0.09

### Assessment of the efficiencies of *Corallina officinalis* Kützing dry biomass for the removal of light n-alkanes, heavy *n*-alkanes, and polycyclic aromatic hydrocarbons (PAHs)

Tables 8, 9, and 10 show that the concentration of the remained light *n*-alkanes in wastewater using *C. officinalis* dry biomass decreased from 51.12 to 20.65 µg/l. Heavy *n*-alkanes remained in wastewater and decreased from 74.7 to 30.69 µg/l after 15 days. PAHs decreased from 21.16 to 6.16 µg/l after 15 days. The alga achieved removal percentages of 59.60%, 58.92%, and 70.88% for light *n*-alkanes, heavy n-alkanes, and PAHs, respectively, after 15 days (Fig. 2).

**Table 8:** Concentration of light *n*-alkanes (µg/l) remained in seawater at different time intervals using *Corallina officinalis* dry biomass

Hydrocarbon conc. (µg/l)				
Hydrocarbon	Time (days)			
	Initial conc. conc	5th day	10th day	15th day
C_13_	6.92 ± 0.49	5.45 ± 0.34	4.69 ± 0.30	2.33 ± 0.20
C_14_	6.45 ± 0.46	5.22 ± 0.31	3.34 ± 0.20	2.11 ± 0.18
C_15_	5.35 ± 0.36	4.28 ± 0.28	2.33 ± 0.43	2.31 ± 0.20
C_16_	4.96 ± 0.29	3.69 ± 0.22	3.35 ± 0.23	2.15 ± 0.18
C_17_	4.88 ± 0.25	3.38 ± 0.24	2.23 ± 0.19	2.01 ± 0.20
Pristane	3.92 ± 0.22	3.12 ± 0.21	2.37 ± 0.19	2.02 ± 0.15
C_18_	5.82 ± 0.37	4.15 ± 0.24	3.29 ± 0.21	2.41 ± 0.21
Phytane	6.55 ± 0.39	4.69 ± 0.29	3.12 ± 0.22	2.61 ± 0.22
C_19_	3.43 ± 0.21	2.80 ± 0.20	2.63 ± 0.20	1.24 ± 0.14
C_20_	2.84 ± 0.18	1.76 ± 0.19	1.36 ± 0.15	1.46 ± 0.14
∑Light *n*-alkanes	51.12 ± 4.25	38.72 ± 4.12	28.71 ± 3.22	20.65 ± 2.65

**Table 9:** Concentration of heavy *n*-alkanes (µg/l) remained in seawater at different time intervals using *Corallina officinalis* dry biomass

Hydrocarbon conc. (µg/l)				
Hydrocarbon	Time (days)			
	Initial conc. conc	5th day	10th day	15th day
C_21_	2.77 ± 0.19	2.32 ± 0.20	2.02 ± 0.16	1.98 ± 0.15
C_22_	2.71 ± 0.19	2.34 ± 0.22	2.12 ± 0.16	1.82 ± 0.15
C_23_	3.38 ± 0.27	2.42 ± 0.22	2.31 ± 0.18	2.02 ± 0.19
C_24_	4.86 ± 0.22	3.38 ± 0.24	3.11 ± 0.21	2.12 ± 0.18
C_25_	5.67 ± 0.33	4.48 ± 0.26	3.23 ± 0.22	2.03 ± 0.18
C_26_	5.45 ± 0.31	4.02 ± 0.20	2.81 ± 0.19	2.11 ± 0.17
C_27_	4.71 ± 0.23	4.11 ± 0.20	3.24 ± 0.22	2.98 ± 0.19
C_28_	5.61 ± 0.33	4.82 ± 0.29	3.64 ± 0.24	2.72 ± 0.19
C_29_	5.21 ± 0.26	4.22 ± 0.24	3.14 ± 0.22	1.64 ± 0.16
C_30_	5.15 ± 0.26	4.23 ± 0.26	3.96 ± 0.26	2.83 ± 0.19
C_31_	6.89 ± 0.35	5.66 ± 0.33	3. 88 ± 0.24	2.32 ± 0.18
C_32_	5.67 ± 0.32	3.42 ± 0.22	3.02 ± 0.21	1.24 ± 0.15
C_33_	4.94 ± 0.25	4.43 ± 0.24	2.34 ± 0.16	1.13 ± 0.15
C_34_	4.92 ± 0.22	3.03 ± 0.20	2.12 ± 0.16	1.23 ± 0.15
C_35_	3.90 ± 0.19	3.42 ± 0.22	2.32 ± 0.19	1.48 ± 0.17
C_36_	2.86 ± 0.17	1.62 ± 0.19	1.24 ± 0.14	1.04 ± 0.14
∑Hteavy *n*-alkanes	74.7 ± 5.12	57.92 ± 4.52	40.62 ± 4.22	30.69 ± 3.51

**Table 10:** Concentration of polycyclic aromatic hydrocarbons (µg/l) remained in wastewater at different time intervals using *Corallina officinalis* dry biomass

Polycyclic aromatic hydrocarbons conc. (µg/l)				
PAHs	Time (days)			
	Initial conc	5th day	10th day	15th day
Naphthalene	4.28 ± 0.29	3.46 ± 0.26	2.11 ± 0.19	1.16 ± 0.36
Flurene	2.47 ± 0.26	2.12 ± 0.29	1.56 ± 0.16	0.73 ± 0.12
Phenanthrene	5.82 ± 0.31	3.98 ± 0.28	2.12 ± 0.18	1.26 ± 0.19
Benzo (a) anthracene	2.52 ± 0.19	2.21 ± 0.19	1.82 ± 0.16	0.84 ± 0.29
Anthracene	2.29 ± 0.17	2.08 ± 0.18	1.36 ± 0.35	1.19 ± 0.39
Pyrene ∑ PAHs	3.87 ± 0.27	2.03 ± 0.0.18	1.75 ± 0.16	0.98 ± 0.28
∑PAHs	21.16 ± 2.75	15.88 ± 2.72	10.72 ± 1.34	6.16 ± 1.14

It is clear from the results that for all three selected algae, hydrocarbon adsorption increased with the increase of contact time since a large decrease was observed after 15 days. The observed decrease of light *n*-alkanes, heavy *n*-alkanes, and PAHs in the wastewater by the three studied species is largely due to the adsorption of these compounds to the algal surface. This suggestion was supported by some previous studies, which documented that most hydrophobic organic compounds (HOCs) tend to bioconcentrate (adsorbed) on algae (Saxena et al. 2021; Du et al. 2022).

*Sargassum latifolium* dry biomass was more potential as adsorbent for light *n*-alkanes, heavy *n*-alkanes, and polycyclic aromatic hydrocarbons from wastewater than *U. intestinalis* and *C. officinalis*. Our results are going in harmony with those of Chung et al. (2007), who reported that the removal percentage of aqueous phenanthrene by *Sargassum* was in the range of 91.7–98.4% dead tissue of brown seaweed.

### Bioadsorption of heavy metals

**Fig. 3:**
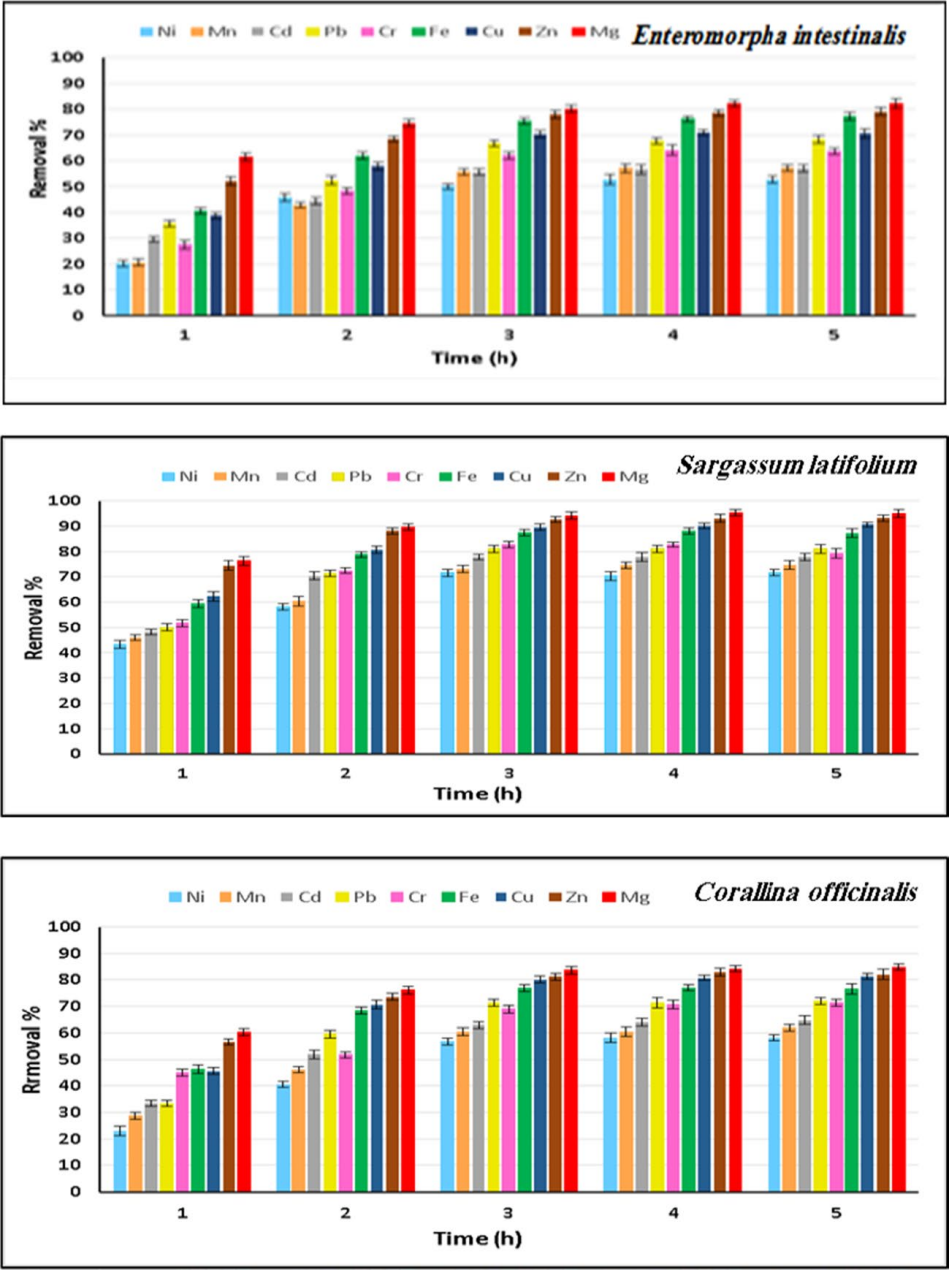
Removal percentage of heavy metals (Ni, Mn, Cd, Pb, Cr, Fe, Cu, Zn, and Mg) from wastewater at different time intervals using *Ulva intestinalis*, *Sargassum latifolium*, and *Corallina officinalis* dry biomass

Heavy metal removal rate from wastewater by algal materials was measured at different time intervals (1–5 h) under natural environmental conditions. All tested heavy metals were influenced by the biological treatment of wastewater, which showed a significant reduction in concentration. As shown in Fig. 3, the removal percentage of heavy metals increased with contact time. The higher removal percentage of all heavy metals by three algal materials is achieved at 3 h, and then an increase in contact time beyond 3 h had a negligible improvement in the removal percentage. The order of metal removal by the three algal materials is as the following: *U. intestinalis* Mg > Zn > Fe > Cu > Pb > Cr > Cd = Mn > Ni, *S. latifolium* Mg > Zn > Cu > Fe > Cr > Pb > Cd > Mn > Ni, *C. officinalis* Mg > Zn > Cu > Fe > Pb > Cr > Cd > Mn > Ni.

*S. latifolium* possessed the highest removal potential for tested metals followed by *C. officinalis* and then *U. intestinali*s which attained the lowermost removal potential. Whereas at 3 h, *S. latifolium* achieved removal percentages of 94.14, 92.62, 89.54, 87.54, 82.76, 80.95, 77.78, 73.02, and 71.62% of Mg, Zn, Cu, Fe, Cr, Pb, Cd, Mn, and Ni, respectively, while *C. officinalis* attained a removal percentage of 83.57, 81.15, 80.13, 76.56, 71.43, 68.96, 62.96, 60.32, and 56.76% of Mg, Zn, Cu, Fe, Pb, Cr, Cd, Mn, and Ni, respectively. *U. intestinalis* removed 80.12, 77.89, 75.40, 70.35, 66.67, 62.07, 55.57, 55.56, and 50% of Mg, Zn, Fe, Cu, Pb, Cr, Cd, Mn, and Ni, respectively, after 3 h.

One of the most important parameters influencing heavy metal removal as well as cost assessments is contact time, especially with the application of the process under natural environmental conditions. Most frequently, the contact time analysis pattern reveals that heavy metal elimination increases early in the process. The adsorption that occurs physically or the exchange of ions that takes place on a solid adsorbent’s surface causes a significant discrepancy between the concentration of heavy metal in the solution and its value on the adsorbent surface. Increasing contact time would have no further impact because there are initially more free-binding sites available before they start to decline and stagnate as a result of a decreasing number of empty sites for binding metal ions on the surface (Redha 2020; Znad et al. 2022). Furthermore, Bai and Venkateswarlu (2018) demonstrated that the biosorption process slows with time, and the adsorbate forms a one-molecule-thick layer on the surface. Indeed, the nonliving algal biomass benefits from the quick adsorption process, making it an effective agent for real-world uses in adsorption units (Mohammed et al. 2019).

Our findings are in conformity with Tabaraki et al. (2014), who found that the optimum contact time for adsorption Pb(II) by *Sargassum ilicifolium* was 200 min, and also Singh (2007) found that the maximum percentage removal of Ni(II) by dried biomass of *Oscillatoria* sp. and *Spirogyra* was achieved at 120 min, while Shyamala Devi et al. (2010) reported that the contact time 240 min was considered the equilibrium time for removing mercury by *Sphaeroplea* dried biomass. In addition, our results agree with El-Sheekh et al. (2022), who reported that with the help of produced silver nanoparticles from *Sargassum latifolium* and its aqueous extract, the adsorption of Fe^+2^ reached equilibrium after 150 min. The initial concentration of metal ions significantly impacts the algal biomass’s ability to biosorb heavy metal ions from aqueous solutions. With an increase in the initial metal ion concentration, the algal biomasses’ biosorption capacity for heavy metal removal improved. This can be due to the stronger driving force that elevated metal ion concentration provides, which beats on mass transfer resistances shown between the biosorbent and the aqueous solution (Rangabhashiyam and Balasubramanian 2018). This may explain why the three algal materials remove metals in the same order as they were initially concentrated in the effluent. Due to its large abundance in wastewater, magnesium takes the top spot among the three algal materials in the removal of metals.

Znad et al. (2022) reported that seaweed has excellent potential for metal removal. The biosorption of various heavy metal ions seems to depend on the type of algae used as well as the conditions under which the processes were conducted. When it comes to macro-algae, the cell walls of brown algae are mostly made of cellulose, alginic acid, polymers (such as mannuronic and guluronic acids) that are complexed with light metals (like sodium, potassium, and calcium), and polysaccharides (e.g., fucoidan). Fucoidan and alginate can bind metals via the exchange of ions, and the primary biosorption binding sites in alginate are carboxyl groups, followed by sulfate groups. Green algal cell walls mostly consist of proteins, which contain functional groups including amino, carboxyl, hydroxyl, and sulfate that aid in metal biosorption. On the other hand, red algae have cell walls primarily made of cellulose, but their ability to absorb nutrients comes from the presence of sulphated polysaccharides made by galactans (He and Chen 2014; Redha 2020).

The results proved that *S. latifolium* has the highest affinity of tested metal adsorption. Brown algae are said to have a higher metal uptake capability than red or green algae, and according to the Shamim (2018) research, many investigations also reported the superiority of brown algae as biosorbents for heavy metals. For instance, *Sargassum wightii* was found suitable for removing arsenic from an aqueous solution (Christobel and Lipton 2015); *Sargassum polycystum* was found by Jayakumar et al. (2021) as an effective biosorbent for cadmium and zinc removal.

### Fourier‑transform infrared spectroscopy (FTIR)

**Fig. 4:**
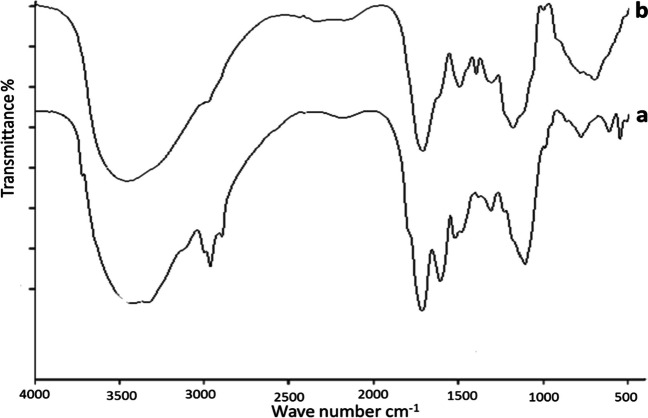
FTIR of *Ulva intestinalis*, before (**b**) and after (**a**) adsorption experiments

**Fig. 5:**
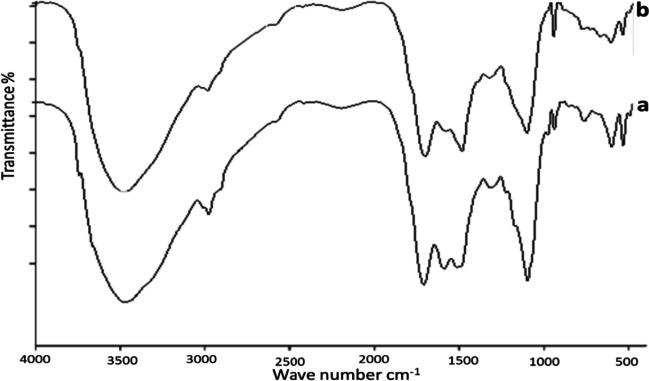
FTIR of *Saragassum latifolium* before (**b**) and after (**a**) adsorption experiments

**Fig. 6:**
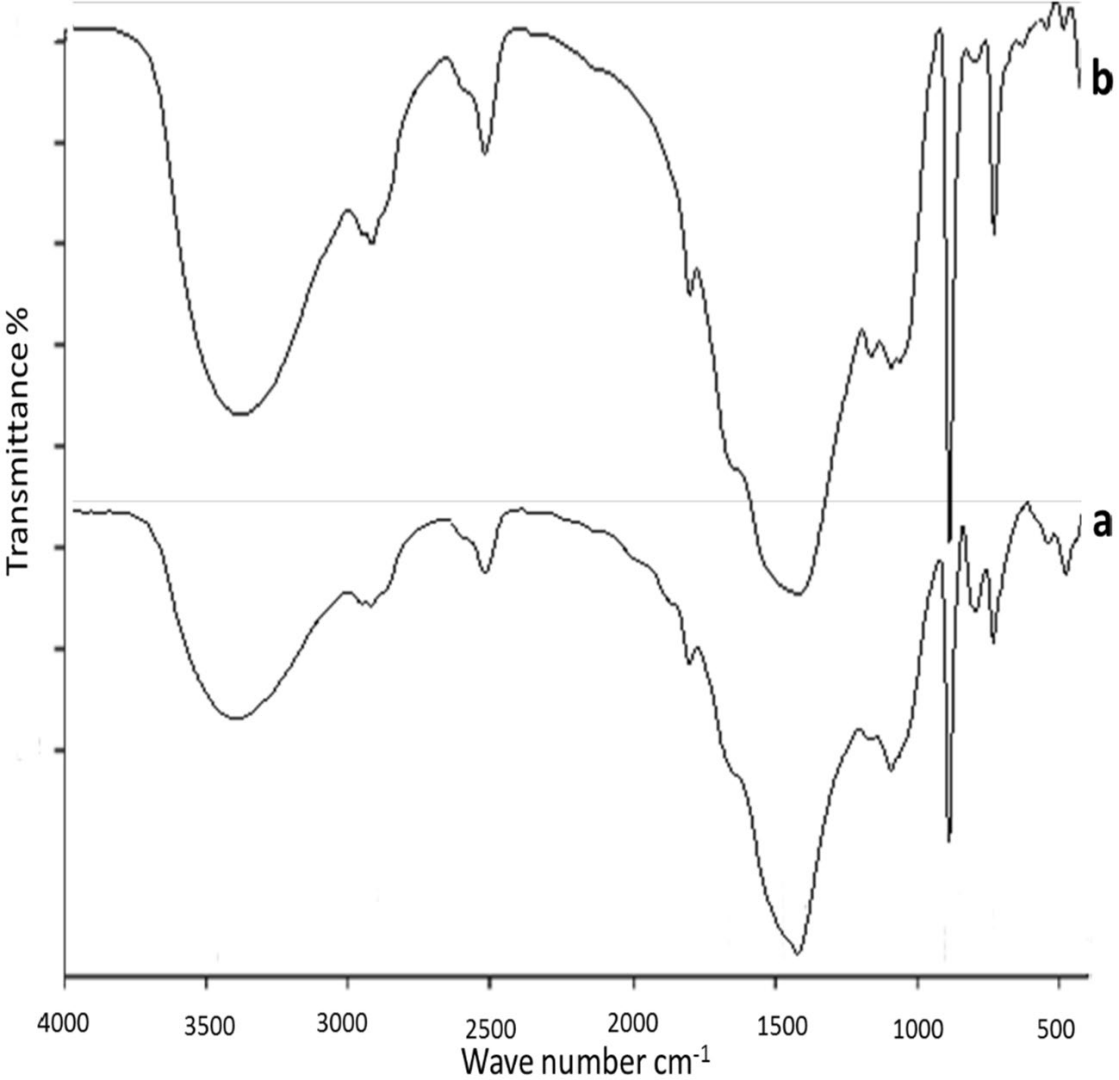
FTIR of *Corallina officinalis* before (**b**) and after (**a**) adsorption experiments

FTIR technique was used to investigate the algal surface, and the three algae were also seen to undergo chemical alteration after wastewater treatment. Identification of the functional groups involved in the biosorption process is made possible through the analysis of the natural and enhanced biomass. Polysaccharides, proteins, and lipids, which are components of the algal cell wall, are the source of these groupings. Numerous functional groups, including carboxyl (such as fatty acids and amino acids), hydroxyl (such as polysaccharides), amine, phosphate, and sulfonate, among others, are available in the cell wall (Dmytryk et al. 2014). In the range of 4000–400 cm^−1^, FTIR spectra of the algae were collected before and after water treatment and are shown in Figs. 4, 5, and 6, which revealed a complex character of the biomass due to the presence of several peaks. After raw biomass and dirty water interacted, the peaks were seen to expand and move. The variations in algal cell wall characteristics, surface area, and surface charge density may cause variations in removal rates between the various species of algae (Gupta and Rastogi 2009). Ion exchange or physical adsorption at the cell surface may be used to remove wastes (Gupta and Rastogi 2009). The findings show that the functional groups on the surface wall of algae and the removed wastes exhibit high affinity and a powerful electrostatic force of attraction (Cengiz and Cavas 2008). Several polysaccharides in the algal cell walls are linked to proteins and other elements (Williams and Edyvean 1997; Tüzün et al. 2005). The functional groups on these molecules, which are present on the surface of algal cells, include carboxyl, amino, phosphate, thiol, and sulfhydryl groups (Tüzün et al. 2005).

The main absorption peaks, referred to as amine N–H and hydroxyl O–H stretching vibrations, show the presence of phenol or alcohol at wavenumbers between 3300 and 3400 cm^−1^ (Mohamed et al. 2021) at 2200–2900 cm^−1^ for all biomasses. –CH stretching vibration of C–CH_3_ occurs. It also referred to N = C = S stretching bond (Lu and Rasco 2012); at ~ 1700–1600 cm^−1^, the peak of C = O stretching mode of the amide I band occurs, and the absorption band of amide I occurs (Demir et al. 2015). The absorption peak at ~ 1400–1500 cm^−1^ represented the stretching of the C = O bond from carboxylic acids (O–H bending) (Younger 2014), in the range 1200–1300 cm^−1^ bending vibrations of C–H and O–H, rocking vibrations of CH_2_ (polysaccharides), and III amide band (proteins) occurs which assigned to cellulose, proteins, lipids, and sulfurous components (ElSaied et al. 2021). The absorption bands at 701–658 cm^−1^ show the stretching of C = S, which suggests sulfides in the tested algae (Touliabah, et al. 2022). The remaining spectra can primarily be attributed to cellulose, aliphatics of the cell wall, polysaccharides and monosaccharides, and aromatic chemicals. According to the FTIR findings, transmittance values were primarily shifted for carboxyl and hydroxyl groups. Additionally, it was observed that discrepancies between the peaks of the treated and untreated algal biomass were greater for *Sargassum latifolium*, which agrees with results obtained by GC, EDX, and SEM.

### EDX

**Fig. 7:**
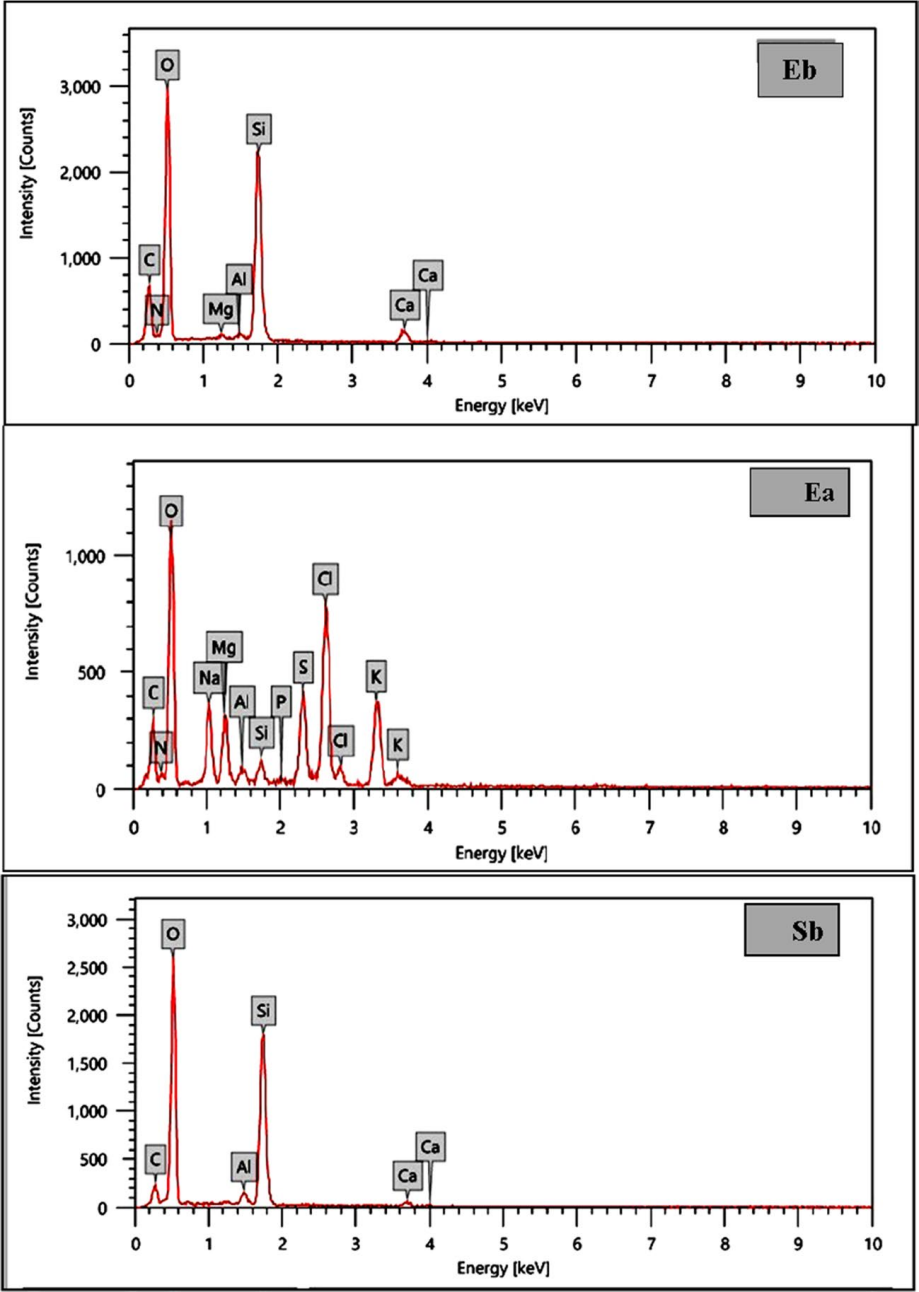

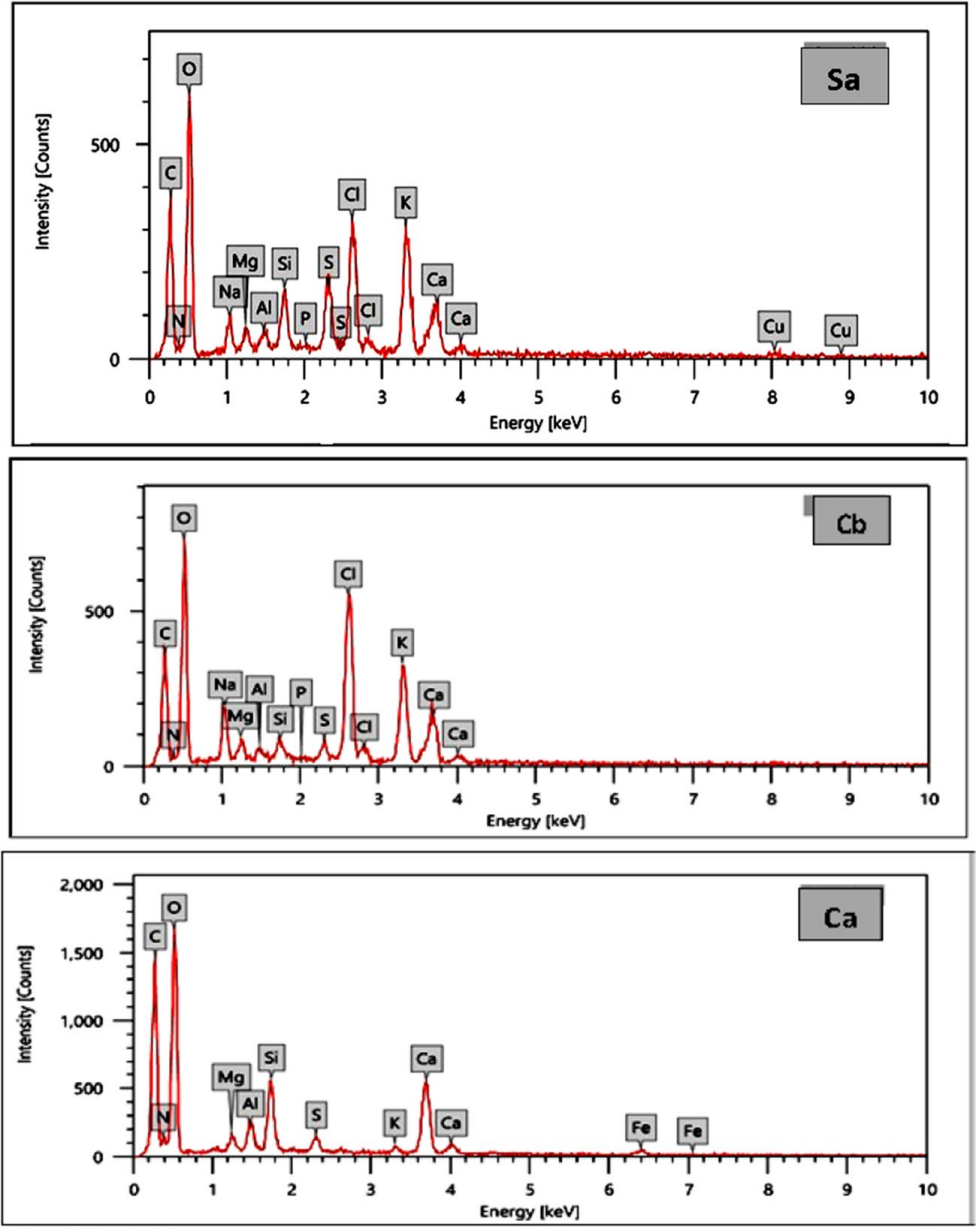
EDX of the surface of *U. intestinalis* (E), *S. latifolium* (S), and *C. officinalis* before (**b**) and after (**a**) adsorption experiments

Using energy-dispersive X-ray (EDX) analysis, the effectiveness of the adsorption of heavy metals by seaweed was further confirmed. Figure 7 shows the typical EDX pattern for three algal materials before and after the sorption of tested heavy metals. It is noteworthy that EDX analysis enables to provide information about the composition of the adsorbent surface, the results (in terms of weight and atomic percentages) demonstrated the presence of C, O, and N, which are the main components of cellular macromolecules (Sultana, et al. 2020). The EDX pattern for the unloaded (native) *U. intestinali*s shows a signal of Mg ion, which increased obviously in metal-loaded *E. intestinali*s. Furthermore, the presence of Ca^+2^, which has been demonstrated participation in the ion exchange process with some metal ions, as indicated in the spectra of unloaded (native) *U. intestinali*s and was not detected in the EDX spectrum of loaded *U. intestinali*s. The EDX pattern for the unloaded (native) *S. latifolium* did not show the characteristic signal of Mg and Cu ions, whereas for the metal-loaded *S. latifolium*, the signals of these ions were observed. The EDX pattern for the unloaded (native) *C. officinalis* did not show the characteristic signal of Fe ion, whereas two signals of this ion were observed in the metal-loaded *C. officinalis*. In addition, the unloaded (native) *S. latifolium* shows a signal of Mg ion, which increased in metal-loaded *S. latifolium*.

### SEM

**Fig. 8:**
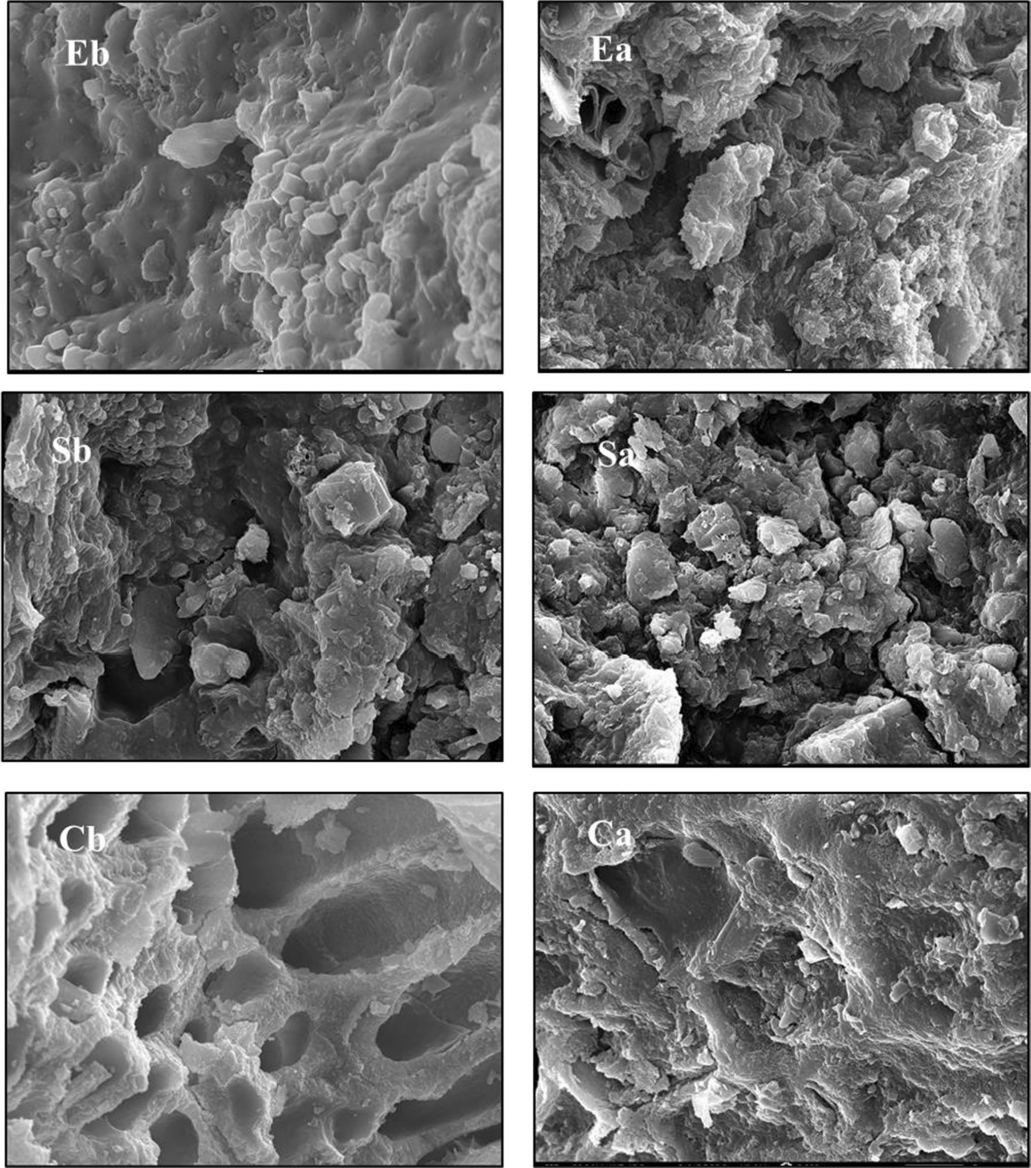
Scanning electron microscopy of the surface of *U. intestinalis* (E), *S. latifolium* (S), and *C. officinalis* before (**b**) and after (**a**) adsorption experiments

The changes in cells’ surface morphology of *U. intestinalis*, *S. latifolium*, and *C. officinalis* biomasses in response to *n*-alkanes, polycyclic aromatic hydrocarbons, and heavy metals adsorption were illustrated via SEM (Fig. 8). Before being exposed to contaminants, algal cells had a smooth, extremely porous structure that was hole-like (Fig. 8, Eb, Sb, and Cb). The surface of the biomass cells became rough and meandering after being exposed to *n*-alkanes, polycyclic aromatic hydrocarbons, and heavy metal ions due to the precipitation of contaminants ions surrounding the cell surface (Fig. 8, Ea, Sa, and Ca), These morphological alterations could be related to variations in the pores, morphology, and structure of algae’s cell walls. The cell wall of *S. latifolium* was very porous and ion permeable. This could explain why *S. latifolium* biomass has the highest affinity for *n*-alkane, polycyclic aromatic hydrocarbon, and heavy metal removal. Omar et al. (2018) and Michalak et al. (2018) observed modifications in algal surface porosity brought and induced by dye adsorption. Christobel and Lipton (2015) reported that SEM micrographs of different algal biomasses illustrate changes in cell morphology due to heavy metal removal.

### Zeta potential (ζ)

**Fig. 9:**
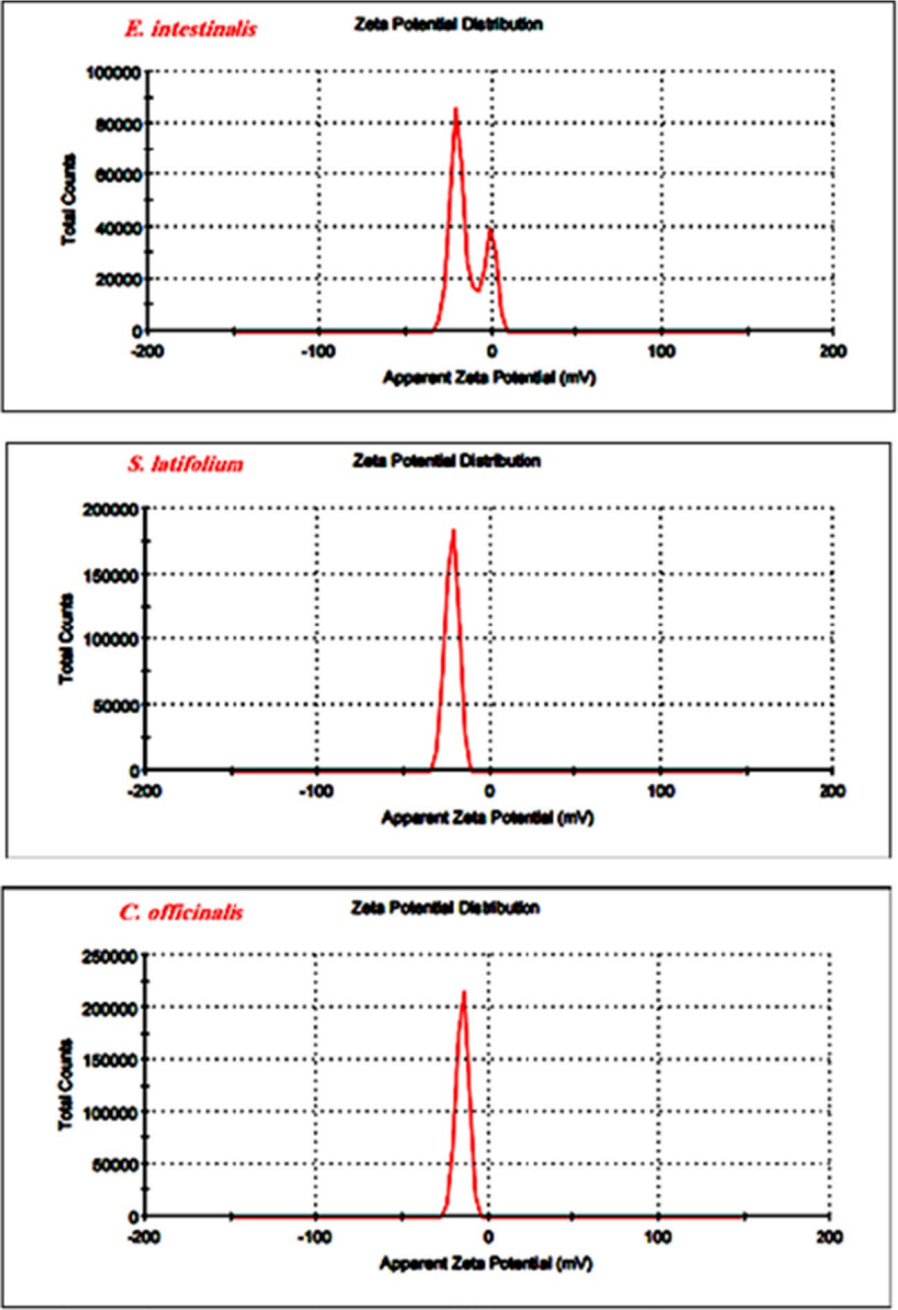
Zeta potential of U. intestinalis, S. latifolium, and C. officinalis

The zeta potentials reveal that algal cells have a charge on the surface (mostly negatively charged) (Taki et al. 2008). Figure 9 shows that the surface charges of *E. intestinalis*, *S. latifolium*, and *C. officinalis* were − 14.3 ± 5.09 mV, − 22.0 ± 3.80 mV, and − 15.1 ± 3.52 mV, respectively; in this study, according to zeta potentials measurements, the cell surface of *S. latifolium* has a more negative surface charge than for *U. intestinalis* and C. officinalis. This agrees with our above gas chromatography-mass spectrometer (GC–MS) results, which showed that *S. latifolium* dry biomass has more potential as an adsorbent for light *n*-alkanes, heavy *n*-alkanes, and polycyclic aromatic hydrocarbons from wastewater than *U. intestinalis* and *C. officinalis*.

## Conclusion

This research highlights the potential of seaweed dry biomasses for the use in bioadsorption of *n*-alkanes, polycyclic aromatic hydrocarbons, and heavy metals from wastewater under natural environmental conditions. Different *n*-alkanes, polycyclic aromatic hydrocarbons, and heavy metals ions were greatly influenced by the biological treatment of wastewater using *U. intestinalis*, *S. latifolium*, and *C. officinalis*, where they showed a high reduction in terms of concentration. The experiments were conducted at natural climatic conditions as a function of contact time. The optimum contact time for *n*-alkanes and polycyclic aromatic hydrocarbons was 15 days, while that for heavy metals removal was 3 h. *S. latifolium* was the best bioadsorbent for *n*-alkanes, polycyclic aromatic hydrocarbons, and heavy metals from wastewater, followed by *C. officinalis* and *U. intestinalis*. According to this study, seaweed biomasses might make a straightforward, environmentally friendly, economically viable, and secure alternative biosorbent for fine-tuning wastewater treatment.

## Data Availability

Data will be made available on request.
